# SieveSifter: a web-based tool for visualizing the sieve analyses of HIV-1 vaccine efficacy trials

**DOI:** 10.1093/bioinformatics/btx168

**Published:** 2017-04-04

**Authors:** Andrew Fiore-Gartland, Nicholas Kullman, Allan C deCamp, Graham Clenaghan, Wayne Yang, Craig A Magaret, Paul T Edlefsen, Peter B Gilbert

**Affiliations:** 1 Vaccine and Infectious Disease Division, Fred Hutchinson Cancer Research Center, Seattle, WA, USA; 2 eScience Institute, University of Washington, Seattle, WA, USA; 3 Department of Biostatistics, University of Washington, Seattle, WA, USA

## Abstract

**Summary:**

Analysis of HIV-1 virions from participants infected in a randomized controlled preventive HIV-1 vaccine efficacy trial can help elucidate mechanisms of partial protection. By comparing the genetic sequence of viruses from vaccine and placebo recipients to the sequence of the vaccine itself, a technique called ‘sieve analysis’, one can identify functional specificities of vaccine-induced immune responses. We have created an interactive web-based visualization and data access tool for exploring the results of sieve analyses performed on four major preventive HIV-1 vaccine efficacy trials: (i) the HIV Vaccine Trial Network (HVTN) 502/Step trial, (ii) the RV144/Thai trial, (iii) the HVTN 503/Phambili trial and (iv) the HVTN 505 trial. The tool acts simultaneously as a platform for rapid reinterpretation of sieve effects and as a portal for organizing and sharing the viral sequence data. Access to these valuable datasets also enables the development of novel methodology for future sieve analyses.

**Availability and Implementation:**

Visualization: http://sieve.fredhutch.org/viz. Source code: https://github.com/nkullman/SIEVE. Data API: http://sieve.fredhutch.org/data.

## 1 Introduction and motivation

A major goal of preventive HIV-1 vaccine efficacy trials is to identify immune correlates of risk and protection (Plotkin and Gilbert, 2012). Identification of vaccine-induced immune responses that correlate with infection can aid in the rational design of novel vaccine candidates. A complementary approach compares the genetic sequences of ‘breakthrough’ viruses that infect vaccine recipients versus those that infect placebo recipients, to identify evidence of vaccine-specific immune pressure (Edlefsen *et al.*, 2013; Gilbert *et al.*, 1998). The underlying hypothesis is that vaccine responses will more effectively target viruses that are similar to the vaccine immunogens. This approach is termed ‘sieve analysis’ with vaccine immune responses acting as a sieve to selectively block transmission of specific viruses. Statistically significant differences between the randomized vaccine and placebo treatment groups generate hypotheses about the vaccine responses that mediate partial protection.

A fundamental challenge in sieve analysis is the identification of potentially small footprints of vaccine-induced pressure (1 AA to ∼50 AA) within an HIV-1 genome of >3000 amino acids. Since HIV-1 vaccine efficacy trials have historically accumulated between 47 and 368 HIV-1 infected cases (mean 142) in each trial, there is limited statistical power to identify sites under selective pressure. Nevertheless, effects were detected in the RV144 trial, which is the only trial to demonstrate partial efficacy of an HIV-1 vaccine (Edlefsen *et al.*, 2015; Rolland *et al.*, 2012). The primary sieve analysis focused on known antibody contact sites in Env V1V2; IgG antibodies targeting these sites were induced by the vaccine and were correlated inversely with HIV-1 infection (Haynes *et al.*, 2012). At one of these sites (HXB2 K169), viruses from vaccine versus placebo recipients were more likely to mismatch the vaccine. Follow-up experiments have shown that mutations at this site abrogate binding of vaccine-elicited V2-specific IgG, thus helping to validate the sieve analysis findings (Zolla-Pazner *et al.*, 2014). The finding demonstrates the importance of leveraging experimental data in sieve analysis; as more data become available, the results of a sieve analysis can gain significance. Significant barriers (e.g. ethical, resources, sociopolitical) make it challenging to reproduce human HIV-1 vaccine efficacy trials to validate hypotheses, making it even more critical to share data widely and ensure that they are fully explored for potential insight.

To facilitate future discovery and a deeper understanding of vaccine responses, we have created a publicly accessible and interactive web-based tool for exploring the sieve analyses of HIV-1 vaccine efficacy trials. The visualization allows for site-specific comparisons of sequences isolated from vaccine versus placebo recipients. At publication the tool includes data and results from the HVTN 502/Step trial (Buchbinder *et al.*, 2008; Rolland *et al.*, 2011), the HVTN 503/Phambili trial (Gray *et al.*, 2011; Hertz *et al.*, 2016), the RV144 Thai trial (Edlefsen *et al.*, 2015; Rerks-Ngarm *et al.*, 2009) and the HVTN 505 trial (Hammer *et al.*, 2013).

## 2 Materials and methods

Sieve analysis is based on a statistical test comparing viral sequences from infected vaccine versus placebo recipients. See primary manuscripts for sequencing details. The unit for each participant is typically a sequence-based genetic distance representing the (dis)similarity of the virus and the vaccine immunogen. One example is a site-specific ‘mismatch’ (i.e. Hamming or ‘VXMatch’) distance that indicates whether or not a breakthrough sequence matches the vaccine at each site. The treatment groups are compared using one of a menu of two-sample statistics and a 2-sided p-value generated by permutation (10K permutations). The tool also provides Benjamini-Hochberg false-discovery rate (FDR) adjusted q-values across all sites in each protein.

## 3 Implementation and usage

### 3.1 Visualization

**Fig. 1:**
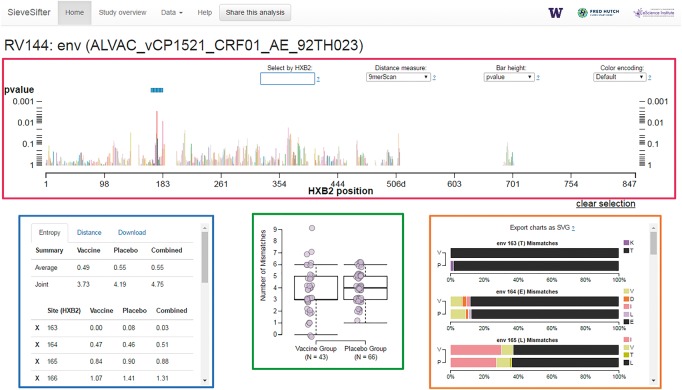
SieveSifter layout. A sieve analysis of a single immunogen/protein for an HIV-1 vaccine efficacy trial is visualized in four primary frames: (**A**, red) a sequence navigator showing the site-specific sieve analysis results (**B**, blue) a summary table of the selected sites, (**C**, green) a summary chart of distributions of vaccine versus placebo group distances over the selected sites and (**D**, orange) stacked bar charts showing the distributions of vaccine versus placebo amino acids at each selected site

The visualization was constructed using HTML5 and Javascript using the D3 library. A tour is available on the site that walks the user through the interface. It has four primary frames (Fig. 1): (A) Sequence navigator showing site-specific sieve analysis results for a single vaccine protein. Results are based on a specific distance measure, which can be selected from the pull-down menu. Each distance captures a different aspect of the dissimilarity between the vaccine reference sequence and the breakthrough viral sequences, which may lead to differing interpretations. A detailed explanation of the distances used for each study are provided in the corresponding sieve analysis manuscript; a summary figure of distance metrics is available in Edlefsen *et al.* (2015). The tool allows sets of sites to be selected. Each bar represents a site and the sequence can be zoomed and panned to move through the data. The height of each bar encodes a selected statistic, including effect magnitude, p-value or q-value, which can be selected using a menu. Residues can be colored by amino acid (default), chemistry, hydrophobicity or Taylor score; (B) Summary table of the selected sites showing entropy and average distance by group; (C) Summary chart showing vaccine versus placebo distances over the selected sites. (D) Stacked bar charts showing the frequencies of amino acids at each selected site. The resulting chart can be exported in Scalable Vector Graphics (SVG) format to generate publication quality figures.

### 3.2 Data application programming interface (API)

The visualization receives data via an API that is also publicly accessible (http://sieve.fredhutch.org/data). Though the HIV-1 sequences from these trials have been deposited in NCBI Genbank, the accessibility of sequence data provided by our portal helps to organize the vaccine immunogen sequences, the whole-genome viral sequences and the associated treatment assignments of the infected participants.
